# Di-μ-chlorido-bis­{[4-chloro-2-(dimethyl­amino­meth­yl)phenyl-κ^2^ *C* ^1^,*N*]palladium(II)}

**DOI:** 10.1107/S1600536810003569

**Published:** 2010-02-03

**Authors:** Yankui Sang, Qiaomin Hong, Wenwei Qiu, Qiancai Liu, Fan Yang

**Affiliations:** a Department of Chemistry, East China Normal University, 3663 North Zhongshan Road, Shanghai 200062, People’s Republic of China

## Abstract

The title compound, [Pd_2_(C_9_H_11_ClN)_2_Cl_2_], consists of two Pd atoms which are bridged by two Cl atoms, forming a centrosymmetric binuclear complex with a square-planar coordination for each of the Pd atoms. The Pd atom is chelated by one N and one C atom from a 4-chloro-2-(dimethyl­amino­meth­yl)phenyl ligand, forming a five-membered ring (N—Pd—C—C—C). In the crystal structure, weak C—H ⋯Cl hydrogen bonds link the mol­ecules in rows.

## Related literature

For cyclo­palladated complexes (CPCs) of tertiary aryl­mines as efficient catalysts in coupling reactions, see: Morales-Morales (2007 ▶); Joshaghani *et al.* (2008 ▶); Xu *et al.* (2009 ▶); Yang *et al.* (2002 ▶); Zheng *et al.* (2003 ▶). For the crystal structures of related CPCs, see: Calmuschi-Cula *et al.* (2005 ▶); Yang *et al.* (2003 ▶); Zhou *et al.* (2010 ▶).
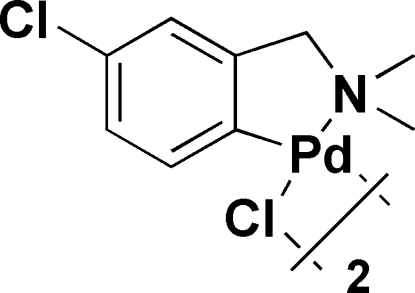


## Experimental

### 

#### Crystal data

[Pd_2_(C_9_H_11_ClN)_2_Cl_2_]
*M* *_r_* = 620.98
Monoclinic, 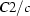

*a* = 28.450 (2) Å
*b* = 5.6325 (5) Å
*c* = 14.2844 (11) Å
β = 111.702 (1)°
*V* = 2126.7 (3) Å^3^
*Z* = 4
Mo *K*α radiation
μ = 2.20 mm^−1^
*T* = 296 K
0.48 × 0.41 × 0.35 mm

#### Data collection

Bruker SMART CCD area-detector diffractometer
Absorption correction: multi-scan (*SADABS*; Bruker, 2000 ▶) *T*_min_ = 0.576, *T*_max_ = 1.000
5903 measured reflections
2315 independent reflections
2173 reflections with *I* > 2σ(*I*)
*R*_int_ = 0.071

#### Refinement

*R*[*F* ^2^ > 2σ(*F* ^2^)] = 0.032
*wR*(*F* ^2^) = 0.087
*S* = 1.10
2315 reflections
119 parameters
1 restraint
H-atom parameters constrained
Δρ_max_ = 0.78 e Å^−3^
Δρ_min_ = −0.59 e Å^−3^

### 

Data collection: *SMART* (Bruker, 2000 ▶); cell refinement: *SAINT* (Bruker, 2000 ▶); data reduction: *SHELXTL* (Sheldrick, 2008 ▶); program(s) used to solve structure: *SHELXS97* (Sheldrick, 2008 ▶); program(s) used to refine structure: *SHELXL97* (Sheldrick, 2008 ▶); molecular graphics: *SHELXTL*; software used to prepare material for publication: *SHELXTL*.

## Supplementary Material

Crystal structure: contains datablocks I, global. DOI: 10.1107/S1600536810003569/bq2191sup1.cif

Structure factors: contains datablocks I. DOI: 10.1107/S1600536810003569/bq2191Isup2.hkl

Additional supplementary materials:  crystallographic information; 3D view; checkCIF report

## Figures and Tables

**Table 1:** Hydrogen-bond geometry (Å, °)

*D*—H⋯*A*	*D*—H	H⋯*A*	*D*⋯*A*	*D*—H⋯*A*
C2—H2⋯Cl2^i^	0.93	2.76	3.283 (4)	117
C9—H9*B*⋯Cl2	0.96	2.77	3.325 (5)	118

Symmetry code: (i) 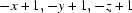.

**Fig. 1.**
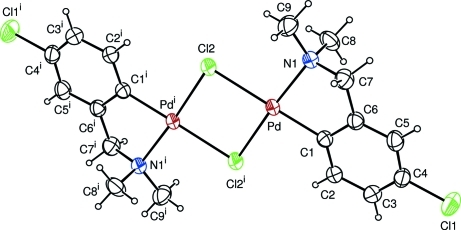
The molecular structure of the title compound, (I), with displacement ellipsoids drawn at 40% probability level. Symmetry code: (i) -x, -y, -z.

**Fig. 2.**
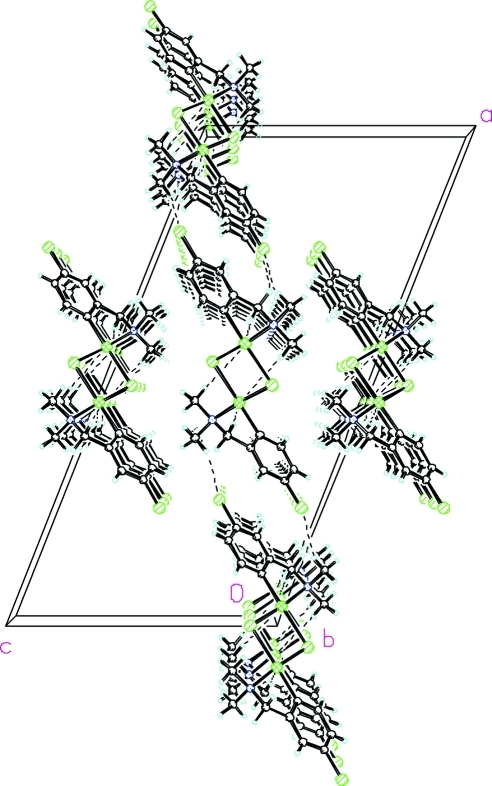
Molecular packing of (I). Dashed lines show H-bonds.

## supplementary crystallographic information

### Comment

Since the discovery of cyclopalladated complexes (CPCs) a half a century ago, these organometallic compounds have found a plethora of applications (Morales-Morales, 2007; Joshaghani *et al.*, 2008; Xu *et al.*, 2009). We have reported the crystal structures of chiral acetate-bridged binuclear cyclopalladated complexes and the application of some cyclopalladated complexes of tertiary arylamines in coupling reactions (Yang *et al.*, 2002; Zheng *et al.*, 2003). In order to compare the catalytic activities of different substituted tertiary arylamine palladacycles at the aromatic ring, we synthesized a series of these compounds by the reaction of 3-substituted *N*,*N*-dimethylbenzylaime with Li_2_PdCl_4_. Herein we report the structure of chloro substituted cyclopalladated complex, Di-µ-chlorobis{4-chloro-2-[(dimethylamino- κ*N*)methyl]phenyl-κ*C*}dipalladium (I).

The two Pd atoms were bridged by two Cl atoms, forming a diamond-planar geometry center (Fig. 1). Each of the two Pd atoms was chelated by one N and one C atoms forming a five-member ring. In the crystal structure, weak C—H···Cl hydrogen bonds link the molecules in rows (Table 1, Fig. 2).

### Experimental

3-Chloro-*N*,*N*-dimethylbenzylamine (3.0 mmol, 0.51 g) and the solution of Li_2_PdCl_4_ (0.26 g, 1.0 mmol) in anhydrous methanol (10 ml) were mixed, and the mixture was stirred for 24 h at room temperature. The reaction mixture was filtered. The yellowish solid was recrystallized with CH_2_Cl_2_ to afford light yellowish crystal.

### Refinement

H atoms were positioned with idealized geometry using a riding model [C—H = 0.93—0.97 Å]. All H atoms were refined with isotropic displacement parameters [set to 1.2 (1.5 for methyl) times of the *U*_eq_ of the parent atom].

### Crystal data

**Table:**

[Pd_2_(C_9_H_11_ClN)_2_Cl_2_]	*F*(000) = 1216
*M**_r_* = 620.98	*D*_x_ = 1.939 Mg m^−^^3^
Monoclinic, *C*2/*c*	Melting point: 473 K
Hall symbol: -C 2yc	Mo *K*α radiation, λ = 0.71073 Å
*a* = 28.450 (2) Å	Cell parameters from 4334 reflections
*b* = 5.6325 (5) Å	θ = 5.1–56.7°
*c* = 14.2844 (11) Å	µ = 2.20 mm^−^^1^
β = 111.702 (1)°	*T* = 296 K
*V* = 2126.7 (3) Å^3^	Prismatic, colourless
*Z* = 4	0.48 × 0.41 × 0.35 mm

### Data collection

**Table:**

Bruker SMART CCD area-detector diffractometer	2315 independent reflections
Radiation source: fine-focus sealed tube	2173 reflections with *I* > 2σ(*I*)
graphite	*R*_int_ = 0.071
phi and ω scans	θ_max_ = 27.0°, θ_min_ = 2.9°
Absorption correction: multi-scan (*SADABS*; Bruker, 2000)	*h* = −36→32
*T*_min_ = 0.576, *T*_max_ = 1.000	*k* = −7→5
5903 measured reflections	*l* = −16→18

### Refinement

**Table:**

Refinement on *F*^2^	Secondary atom site location: difference Fourier map
Least-squares matrix: full	Hydrogen site location: inferred from neighbouring sites
*R*[*F*^2^ > 2σ(*F*^2^)] = 0.032	H-atom parameters constrained
*wR*(*F*^2^) = 0.087	*w* = 1/[σ^2^(*F*_o_^2^) + (0.0385*P*)^2^ + 2.2145*P*] where *P* = (*F*_o_^2^ + 2*F*_c_^2^)/3
*S* = 1.10	(Δ/σ)_max_ = 0.003
2315 reflections	Δρ_max_ = 0.78 e Å^−^^3^
119 parameters	Δρ_min_ = −0.59 e Å^−^^3^
1 restraint	Extinction correction: *SHELXL97* (Sheldrick, 2008), Fc^*^=kFc[1+0.001xFc^2^λ^3^/sin(2θ)]^-1/4^
Primary atom site location: structure-invariant direct methods	Extinction coefficient: 0.00136 (18)

### Special details

**Table:**

Geometry. All esds (except the esd in the dihedral angle between two l.s. planes) are estimated using the full covariance matrix. The cell esds are taken into account individually in the estimation of esds in distances, angles and torsion angles; correlations between esds in cell parameters are only used when they are defined by crystal symmetry. An approximate (isotropic) treatment of cell esds is used for estimating esds involving l.s. planes.
Refinement. Refinement of *F*^2^ against ALL reflections. The weighted *R*-factor wR and goodness of fit *S* are based on *F*^2^, conventional *R*-factors *R* are based on *F*, with *F* set to zero for negative *F*^2^. The threshold expression of *F*^2^ > σ(*F*^2^) is used only for calculating *R*-factors(gt) etc. and is not relevant to the choice of reflections for refinement. *R*-factors based on *F*^2^ are statistically about twice as large as those based on *F*, and *R*- factors based on ALL data will be even larger.

### Fractional atomic coordinates and isotropic or equivalent isotropic displacement parameters (Å^2^)

**Table:**

	*x*	*y*	*z*	*U*_iso_*/*U*_eq_	
C1	0.61584 (12)	0.7531 (6)	0.6472 (2)	0.0377 (6)	
C2	0.63683 (13)	0.6229 (7)	0.7349 (3)	0.0451 (7)	
H2	0.6217	0.4815	0.7422	0.054*	
C3	0.68036 (14)	0.7007 (7)	0.8124 (3)	0.0504 (9)	
H3	0.6943	0.6131	0.8715	0.061*	
C4	0.70230 (13)	0.9093 (7)	0.8000 (3)	0.0475 (8)	
C5	0.68411 (13)	1.0366 (6)	0.7124 (3)	0.0476 (8)	
H5	0.7005	1.1737	0.7046	0.057*	
C6	0.64054 (12)	0.9577 (6)	0.6348 (2)	0.0402 (7)	
C7	0.61785 (14)	1.0829 (6)	0.5364 (3)	0.0482 (8)	
H7A	0.5963	1.2114	0.5418	0.058*	
H7B	0.6443	1.1494	0.5168	0.058*	
C8	0.62160 (16)	0.7642 (8)	0.4257 (3)	0.0571 (10)	
H8A	0.6451	0.6797	0.4819	0.086*	
H8B	0.6398	0.8666	0.3973	0.086*	
H8C	0.6019	0.6530	0.3756	0.086*	
C9	0.55179 (15)	1.0332 (8)	0.3720 (3)	0.0621 (10)	
H9A	0.5294	1.1284	0.3928	0.093*	
H9B	0.5325	0.9194	0.3228	0.093*	
H9C	0.5701	1.1335	0.3431	0.093*	
N1	0.58771 (10)	0.9079 (5)	0.4600 (2)	0.0394 (6)	
Cl1	0.75511 (4)	1.0154 (2)	0.89840 (8)	0.0736 (3)	
Cl2	0.47788 (3)	0.5844 (2)	0.38205 (6)	0.0541 (3)	
Pd	0.554347 (8)	0.67878 (4)	0.530312 (17)	0.03578 (13)	

### Atomic displacement parameters (Å^2^)

**Table:**

	*U*^11^	*U*^22^	*U*^33^	*U*^12^	*U*^13^	*U*^23^
C1	0.0328 (15)	0.0447 (15)	0.0372 (16)	−0.0005 (14)	0.0147 (13)	−0.0023 (14)
C2	0.0404 (17)	0.0540 (18)	0.0401 (18)	−0.0104 (16)	0.0141 (15)	0.0013 (16)
C3	0.0437 (19)	0.066 (2)	0.0407 (19)	−0.0012 (17)	0.0142 (16)	0.0021 (16)
C4	0.0343 (15)	0.062 (2)	0.0442 (19)	−0.0047 (16)	0.0128 (14)	−0.0132 (17)
C5	0.0427 (17)	0.0464 (17)	0.055 (2)	−0.0107 (15)	0.0196 (15)	−0.0109 (16)
C6	0.0388 (15)	0.0407 (16)	0.0419 (17)	−0.0043 (14)	0.0160 (13)	−0.0042 (13)
C7	0.0504 (19)	0.0382 (16)	0.054 (2)	−0.0067 (16)	0.0172 (16)	0.0016 (15)
C8	0.057 (2)	0.067 (2)	0.062 (2)	−0.006 (2)	0.040 (2)	−0.006 (2)
C9	0.054 (2)	0.065 (2)	0.055 (2)	−0.005 (2)	0.0054 (18)	0.0188 (19)
N1	0.0345 (13)	0.0446 (14)	0.0395 (14)	−0.0032 (12)	0.0143 (11)	0.0020 (12)
Cl1	0.0546 (5)	0.0955 (8)	0.0562 (6)	−0.0190 (6)	0.0036 (5)	−0.0209 (6)
Cl2	0.0381 (4)	0.0759 (6)	0.0446 (4)	−0.0177 (4)	0.0109 (3)	0.0122 (4)
Pd	0.02757 (16)	0.04355 (18)	0.03759 (18)	−0.00378 (9)	0.01365 (12)	0.00214 (9)

### Geometric parameters (Å, °)

**Table:**

C1—C2	1.382 (5)	C7—H7B	0.9700
C1—C6	1.395 (5)	C8—N1	1.474 (5)
C1—Pd	1.966 (3)	C8—H8A	0.9600
C2—C3	1.392 (5)	C8—H8B	0.9600
C2—H2	0.9300	C8—H8C	0.9600
C3—C4	1.373 (5)	C9—N1	1.475 (4)
C3—H3	0.9300	C9—H9A	0.9600
C4—C5	1.367 (5)	C9—H9B	0.9600
C4—Cl1	1.740 (3)	C9—H9C	0.9600
C5—C6	1.395 (5)	N1—Pd	2.068 (3)
C5—H5	0.9300	Cl2—Pd^i^	2.3356 (9)
C6—C7	1.490 (5)	Cl2—Pd	2.4683 (9)
C7—N1	1.485 (4)	Pd—Cl2^i^	2.3356 (9)
C7—H7A	0.9700		
			
C2—C1—C6	119.0 (3)	N1—C8—H8B	109.5
C2—C1—Pd	127.4 (3)	H8A—C8—H8B	109.5
C6—C1—Pd	113.6 (2)	N1—C8—H8C	109.5
C1—C2—C3	120.9 (3)	H8A—C8—H8C	109.5
C1—C2—H2	119.6	H8B—C8—H8C	109.5
C3—C2—H2	119.6	N1—C9—H9A	109.5
C4—C3—C2	118.7 (3)	N1—C9—H9B	109.5
C4—C3—H3	120.7	H9A—C9—H9B	109.5
C2—C3—H3	120.7	N1—C9—H9C	109.5
C5—C4—C3	122.1 (3)	H9A—C9—H9C	109.5
C5—C4—Cl1	118.8 (3)	H9B—C9—H9C	109.5
C3—C4—Cl1	119.2 (3)	C8—N1—C9	108.1 (3)
C4—C5—C6	119.0 (3)	C8—N1—C7	109.7 (3)
C4—C5—H5	120.5	C9—N1—C7	109.8 (3)
C6—C5—H5	120.5	C8—N1—Pd	107.0 (2)
C1—C6—C5	120.3 (3)	C9—N1—Pd	114.5 (2)
C1—C6—C7	116.6 (3)	C7—N1—Pd	107.56 (19)
C5—C6—C7	123.1 (3)	Pd^i^—Cl2—Pd	94.18 (3)
N1—C7—C6	108.1 (3)	C1—Pd—N1	81.70 (12)
N1—C7—H7A	110.1	C1—Pd—Cl2^i^	94.72 (10)
C6—C7—H7A	110.1	N1—Pd—Cl2^i^	176.12 (8)
N1—C7—H7B	110.1	C1—Pd—Cl2	179.19 (9)
C6—C7—H7B	110.1	N1—Pd—Cl2	97.75 (8)
H7A—C7—H7B	108.4	Cl2^i^—Pd—Cl2	85.82 (3)
N1—C8—H8A	109.5		
			
C6—C1—C2—C3	3.6 (5)	C2—C1—Pd—N1	−159.6 (3)
Pd—C1—C2—C3	−178.9 (3)	C6—C1—Pd—N1	18.0 (2)
C1—C2—C3—C4	−0.3 (6)	C2—C1—Pd—Cl2^i^	18.9 (3)
C2—C3—C4—C5	−3.1 (6)	C6—C1—Pd—Cl2^i^	−163.5 (2)
C2—C3—C4—Cl1	177.2 (3)	C2—C1—Pd—Cl2	−112 (7)
C3—C4—C5—C6	3.0 (5)	C6—C1—Pd—Cl2	65 (8)
Cl1—C4—C5—C6	−177.3 (3)	C8—N1—Pd—C1	87.3 (3)
C2—C1—C6—C5	−3.8 (5)	C9—N1—Pd—C1	−152.8 (3)
Pd—C1—C6—C5	178.4 (2)	C7—N1—Pd—C1	−30.5 (2)
C2—C1—C6—C7	176.5 (3)	C8—N1—Pd—Cl2^i^	64.6 (13)
Pd—C1—C6—C7	−1.4 (4)	C9—N1—Pd—Cl2^i^	−175.6 (11)
C4—C5—C6—C1	0.5 (5)	C7—N1—Pd—Cl2^i^	−53.2 (13)
C4—C5—C6—C7	−179.7 (3)	C8—N1—Pd—Cl2	−92.1 (2)
C1—C6—C7—N1	−24.2 (4)	C9—N1—Pd—Cl2	27.8 (3)
C5—C6—C7—N1	156.0 (3)	C7—N1—Pd—Cl2	150.1 (2)
C6—C7—N1—C8	−79.8 (3)	Pd^i^—Cl2—Pd—C1	131 (8)
C6—C7—N1—C9	161.5 (3)	Pd^i^—Cl2—Pd—N1	178.46 (8)
C6—C7—N1—Pd	36.3 (3)	Pd^i^—Cl2—Pd—Cl2^i^	0.0

Symmetry codes: (i) −*x*+1, −*y*+1, −*z*+1.

### Hydrogen-bond geometry (Å, °)

**Table:**

*D*—H···*A*	*D*—H	H···*A*	*D*···*A*	*D*—H···*A*
C2—H2···Cl2^i^	0.93	2.76	3.283 (4)	117
C9—H9B···Cl2	0.96	2.77	3.325 (5)	118

Symmetry codes: (i) −*x*+1, −*y*+1, −*z*+1.
